# A Versatile 3D‐Confined Self‐Assembly Strategy for Anisotropic and Ordered Mesoporous Carbon Microparticles

**DOI:** 10.1002/advs.202202394

**Published:** 2022-07-03

**Authors:** Mian Wang, Xi Mao, Jingye Liu, Bite Deng, Shuai Deng, Shaohong Jin, Wang Li, Jiang Gong, Renhua Deng, Jintao Zhu

**Affiliations:** ^1^ State Key Laboratory of Materials Processing and Die & Mould Technology Key Laboratory of Material Chemistry for Energy Conversion and Storage of Ministry of Education School of Chemistry and Chemical Engineering Huazhong University of Science and Technology Wuhan 430074 China

**Keywords:** 3D‐confined self‐assembly, block copolymers, carbon spheres, mesoporous materials, molecular sieves, polystyrene‐block‐poly(4‐vinylpyridine)

## Abstract

Mesoporous carbon microparticles (MCMPs) with anisotropic shapes and ordered structures are attractive materials that remain challenging to access. In this study, a facile yet versatile route is developed to prepare anisotropic MCMPs by combining neutral interface‐guided 3D confined self‐assembly (3D‐CSA) of block copolymer (BCP) with a self‐templated direct carbonization strategy. This route enables pre‐engineering BCP into microparticles with oblate shape and hexagonal packing cylindrical mesostructures, followed by selective crosslinking and decorating of their continuous phase with functional species (such as platinum nanoparticles, Pt NPs) via in situ growth. To realize uniform in situ growth, a “guest exchange” strategy is proposed to make room for functional species and a pre‐crosslinking strategy is developed to preserve the structural stability of preformed BCP microparticles during infiltration. Finally, Pt NP‐loaded MCMPs are derived from the continuous phase of BCP microparticles through selective self‐templated direct carbonization without using any external carbon source. This study introduces an effective concept to obtain functional species‐loaded and *N*‐doped MCMPs with oblate shape and almost hexagonal structure (*p6mm*), which would find important applications in fuel cells, separation, and heterogeneous catalysis.

## Graphical abstract

This study provides a facile yet versatile 3D confined self‐assembly and self‐templating direct carbonation strategy that enables the generation of composite mesoporous carbon microparticles integrating features of anisotropic shape, highly ordered and oriented pore pathways, and uniform and tunable pore size, as well as functional inorganic decoration and *N* doping.

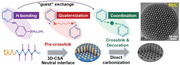

## 1 Introduction

Inorganic mesoporous microparticles integrating the advantages of mesoporous materials with colloidal particulate materials are of critical importance for a range of applications.^[^ ^1^ ^]^ Particularly, mesoporous carbon microparticles (MCMPs)^[^ ^2^ ^]^ and their composites^[^ ^3^ ^]^ are of great interest because of the low density, chemical inertness, conductivity, hydrophobic property, and good biocompatibility of carbon and their wide applications in fuel cells, catalytic cracking of petroleum, separation, drug carriers, and so on.^[^ ^4^ ^]^ In this respect, lots of attention has been given to developing methods for the controllable synthesis of MCMPs with various morphological/structural features, including anisotropic shape,^[^ ^5^ ^]^ ordered structure,^[^ ^2^, ^4^ ^]^ oriented and straight pore channels, and larger pore size.^[^ ^6^ ^]^ Oriented and straight pore channels with larger pore sizes can contribute to mass transportation by smoothing the diffusion pathways. Moreover, MCMPs with large pores show promising applications in large molecule (or nanoparticle, NP) catalysis, separation or delivery, such as petroleum, proteins, and DNA.^[^ ^6^ ^]^ Shape control is also a critical factor. Shape anisotropy could add a new dimension to MCMPs, thereby creating remarkable physical, chemical, and biological properties that cannot be attained by conventional isotropic materials.^[^ ^5^, ^7^ ^]^ Shape anisotropy enables uniform distribution (because the coffee‐ring effect could be prevented) and could also guide the orientation of MCMPs onto targeted substrates (because their rotation was suppressed), as well as exhibit higher adhesive ability.^[^ ^8^ ^]^ In particular, guiding the orientation of MCMPs with pore channels parallel to the mass transmission direction (e.g., membrane electrode and column) would provide attractive high‐efficiency mass transportation while remaining a challenge.

Block copolymers (BCPs) are well known for their controllable self‐assembly and can generate various ordered nanostructures, most of which are on the mesoscale,^[^ ^9^ ^]^ thereby being widely used as structure‐direct agents for the preparation of inorganic mesoporous materials.^[^ ^10^ ^]^ Compared with small molecular surfactants,^[^ ^11^ ^]^ BCPs show great advantages in the construction of large mesopores, as well as flexible control over pore architecture, including shape, size and orientation.^[^ ^10b^ ^]^ In the last decade, significant progress has been achieved in the synthesis of MCMPs and their composites by using BCPs as structure‐direct agents.^[^ ^12^ ^]^ BCP‐directed organic–organic coassembly (with external carbon sources) in solution acts as one of the most popular routes for the preparation of MCMPs. Unfortunately, many solution coassembly methods are not suitable for high‐molecular‐weight BCPs due to the finite solubility, yet inherent size limitations of low molecular weight BCPs (e.g., F127) make them less able to make mesopores >5 nm. Moreover, it is difficult to achieve shape‐anisotropic MCMPs by solution coassembly because spherical particles are the most common outcome as a result of spontaneous minimum interfacial energy. In terms of anisotropic shape, an additional template and specific control of dynamics are required.^[^ ^5b^ ^]^ In addition, due to the collapse of the carbon source during carbonization, it is hard to obtain MCMPs with large, ordered and uniform mesopores.

Compared with solution coassembly, three‐dimensional confined self‐assembly (3D‐CSA) of BCPs shows great advantages in the generation of various shape‐anisotropic microparticles with highly ordered and tunable mesostructures.^[^ ^13^ ^]^ In recent years, a phase separation‐induced 3D confined coassembly method was developed, which provides a way to prepare mesoporous microparticles with anisotropic shapes and large pores.^[^ ^7^, ^14^ ^]^ For example, oblate mesoporous microparticles of aluminosilicate with a hexangular structure (*p6mm*) were obtained. In the preparation of MCMPs, however, this complex coassembly system makes it more difficult to control the self‐assembled morphology because it involves multiple interactions between amphiphilic BCP direct agents, the carbon precursor and another precursor of aluminosilicate/silica, and the dynamics of coassembly and hydrolysis/coagulation. Most recently, emulsion‐solvent evaporation‐driven 3D confined coassembly was applied to prepare MCMPs,^[^ ^15^ ^]^ including anisotropic ones.^[^ ^16^ ^]^ However, methods for the preparation of anisotropic MCMPs with large, ordered and uniform mesopores have rarely been reported.

**Scheme 1:**
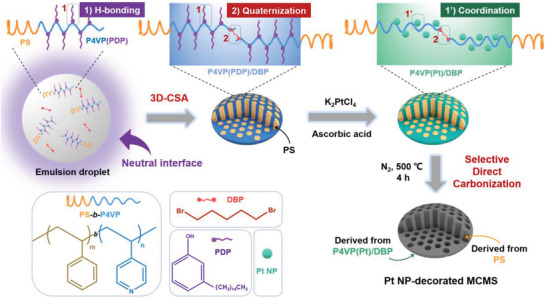
Schematic illustration of the synthesis route of Pt NP‐decorated MCMPs.

Herein, we developed a facile yet versatile route, which allows the generation of composite MCMPs with oblate shape and almost *p6mm* structure (**Scheme** **1**). Polymer microparticles (templates) were firstly pre‐engineered by neutral interface‐guided 3D‐CSA of BCP, polystyrene‐*block*‐poly(4‐vinyl pyridine) (PS‐*b*‐P4VP). In the absence of any external carbon source, 3D‐CSA enables engineering BCP microparticles with ordered mesostructures. The continuous phase (P4VP) of preformed BCP microparticles was then crosslinked and decorated with functional inorganics (e.g., Pt NPs) via in situ growth. A challenge in the liquid phase crosslink is that BCP microparticles will be swelled or even disassembled during infiltration with the solution of crosslinker. To address this challenge, a combination of a comb‐coil supramolecular strategy^[^ ^17^ ^]^ and a pre‐crosslink strategy was proposed. The hydrogen‐bond donor (3‐pentadecyl phenol, PDP) of the supramolecule can be easily removed from the continuous phase due to the reversible weak interaction (i.e., hydrogen bond), making room for subsequent absorption of crosslinker (Pt precursors) via “guest” exchange. The pre‐crosslink strategy can preserve the disassembly of preformed BCP microparticles due to the strong quaternization between P4VP and the pre‐crosslinker (1, 5‐dibromopentane, DBP). Finally, composite MCMPs are derived from the crosslinked continuous phase through selective self‐templated direct carbonization. Apart from Pt NP‐loaded MCMPs, the concept is versatile for the fabrication of anisotropic mesoporous microparticles with other compositions, such as TiO_2_/C, SiO_2_/C, TiO_2_ and SiO_2_.

## 2 Results and Discussion

### 2.1 BCP Microparticles (Templates) by Neutral Interface‐Guided 3D‐CSA

**Figure 1:**
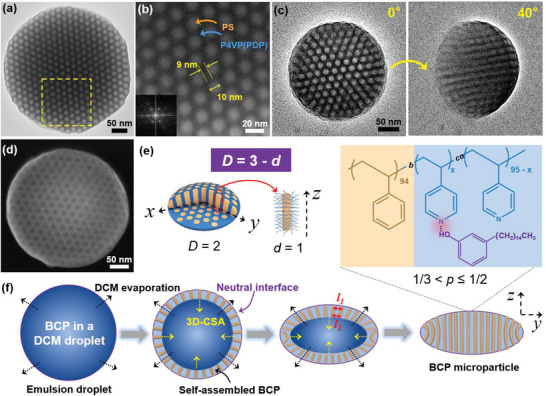
a,b) TEM, c) tilt angle TEM, and d) SEM images of microparticles of PS_94_‐b‐P4VP_95_(PDP)_0.6_/(DBP)_0.4_, the inset of (b) is the FFT image; e) 3D cartoon of the particle. f) Schematic illustration showing the formation process of a particle (DBP is not shown), where cross‐sections of the droplet and the derived particle are shown.

In this study, the supramolecular strategy is combined with the neutral interface strategy to shape targeted microparticles by emulsion‐solvent evaporation‐induced 3D‐CSA of PS‐*b*‐P4VP(PDP) (**Figure** **1**). PS‐*b*‐P4VP is chosen because of the following advantages: 1) P4VP itself can serve as a nitrogen‐doped carbon source after crosslinking, while PS can serve as a pore‐forming agent. We note that the relatively high thermal stability of PS (compared with oxygen‐containing polypropylene oxide, PPO) can support the mesostructure of the particles at the early stage of calcination, which favors the formation of ordered pores. 2) The reactivity of P4VP is much higher than that of PS, allowing selective absorption of functional inorganic precursors. 3) Reactive P4VP can form hydrogen bonds with PDP, which enables flexible tuning of the shape and mesostructures of microparticles. In addition, PDP can be easily removed from the P4VP continuous phase due to the reversibly weak hydrogen bonding interaction, which could make room for subsequent absorption of functional inorganic precursors. The shape and internal structure of targeted microparticles of PS_94_‐b‐P4VP_95_(PDP)_0.6_ in the presence of DBP were investigated, where the content (subscript) of PDP was calculated according to their stoichiometric ratio with respect to pyridine rings of P4VP (*x/n*, *n* = 95). A highly ordered hexangular structure is observed in the bright field TEM images (Figure 1a,b and Figure S1a, Supporting Information), where the darker continuous phase represents the P4VP(PDP)/DBP phase due to its staining with iodine, while the gray hexangular microdomains are composed of PS. The inset fast Fourier transform (FFT) image of the TEM image (Figure 1b) confirms the highly ordered structure of the particles. The same structure is also observed from the SEM image (Figure 1d), where the iodine‐stained P4VP(PDP)/DBP continuous phase looks brighter than the PS microdomains. By combining tilt angle TEM images (Figure 1c), the AFM image and its height profile (Figure S1b,c, Supporting Information), and the top‐view and the side‐view SEM images (Figure S1d,f, Supporting Information), it is clear that they are oblate particles with PS cylinders along the axis. The diameters of the PS cylinders are ≈10 nm, and their distance is ≈9 nm (Figure 1b). DLS data indicated that the average size of the particles was 343.5 nm, and their size distribution was PDI = 0.078 (Figure S2, Supporting Information). In addition, the presence of hydrogen bond (between P4VP and PDP) and quaternarization (between P4VP and DBP) interactions were confirmed by FT‐IR spectroscopy investigation (Figure S3, Supporting Information).

The formation of such unique microparticles is due to the synergistic effect of the deformable 3D soft confinement space and neutral interface interaction (the wettability of both blocks with the confined interface are evenly matched). As shown in Figure 1f, the initial droplet tends to form a spherical shape to minimize the free energy. Then, upon solvent evaporation, the shrinkage of the droplet increases the concentration of PS‐*b*‐P4VP(PDP) and drives their self‐assembly into a shell with a cylinder nanostructure starting from the oil/water interface. Since the interface is neutral wetting with both blocks, all polymer chains are oriented parallel to the interface, resulting in cylinders perpendicular to the interface. The ordered self‐assembly of polymer chains dominates cylinders close‐packing into hexangular nanostructure, which drives deformation of the spherical droplet into oblate shape along with the growth of cylinders from outside‐in (the structural period *l* kept nearly unchanged, *l*_1_ ≈ *l*_2_). Therefore, an oblate particle with a hexangular cylinder nanostructure is finally obtained after the solvent is removed. A more detailed theoretical discussion can be found in a recent report.^[^ ^5d^ ^]^ This mechanism also works for ellipsoid‐ or spherical‐microparticles from lamellae‐ or spherical‐forming BCPs, respectively (Figure S4, Supporting Information). We propose a universal law (*D* = 3 – *d*) for neutral interface‐guided hierarchical self‐assembly of BCPs into anisotropic microparticles under 3D soft confinement (Scheme S1, Supporting Information). *D* and *d* represent the dimensions of microparticles and corresponding building blocks (similar to micelles), respectively. The *D* value equals the packing directions of the building blocks, where the packing direction is dependent on *d*. For example, *D* = 2 for the oblate particle and *d* = 1 for its cylinder building units (Figure 1e). Although BCP microparticles with similar oblate shapes and *p6mm* structures have been reported, they are inverted assemblies with PS as the continuous phase (Scheme S2, Supporting Information).^[^ ^13n^ ^]^ Only individual inorganic nanoobjects or disordered mesoporous particles are accessible by using inverted assemblies as templates because of the pyrolysis of their continuous phase frame.^[^ ^18^ ^]^ To the best of our knowledge, microparticles with the same morphology and composition as shown in this study have not been reported until now.

### 2.2 MCMPs Derived from Composite BCP Microparticles via a Self‐Templated Direct Carbonization Strategy

**Figure 2:**
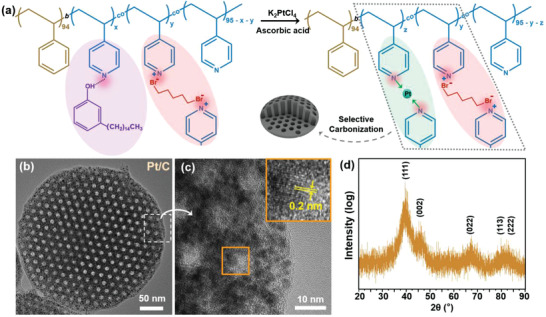
a) Schematic illustration of the in situ decoration of Pt NPs in the continuous phase of BCP microparticles by combining the “guest” exchange strategy and pre‐crosslinking strategy, followed by selective direct carbonization of the continuous phase to achieve Pt NP‐loaded MCMPs and their b,c) HRTEM images, d) wide‐angle XRD spectrum of Pt NPs in MCMPs.

A key feature of the PS‐*b*‐P4VP(PDP) microparticles in the current study is that their continuous phase, which contains reactive pyridine groups, can be selectively crosslinked with functional species, such as Pt NPs. At the meantime, their morphology can be well maintained during infiltration with the solution of Pt NP precursor because of pre‐crosslink. To load Pt NPs throughout the P4VP frame, the absorption of the Pt precursor (K_2_PtCl_4_) was conducted in a mixture of water/ethanol (volume ratio 1:1) because water is a good solvent for K_2_PtCl_4,_ while ethanol is a good solvent for P4VP and PDP. The weak hydrogen bonds between P4VP and PDP can be easily replaced by coordination interactions between P4VP and the Pt precursor, so‐called “guest” exchange (**Figure** **2**a), which is confirmed by FT‐IR (Figure S3, Supporting Information). The release of PDP from BCP microparticles in the mixture of water/ethanol was detected by UV–vis spectroscopy (Figure S5, Supporting Information). After in situ reduction, composite microparticles with Pt NPs located in the P4VP continuous phase were obtained, and the morphology of the microparticles remained unchanged (Figure S6b, Supporting Information), confirming their good structural stability after pre‐crosslinking of P4VP with DBP. In a control experiment, disassembly of BCP microparticles was observed in the absence of DBP (Figure S6d, Supporting Information). We note that the addition of the crosslinker (DBP) has no impact on the morphology of BCP microparticles (Figure S6a,c, Supporting Information).

The loading ratio of Pt NPs plays a critical role in determining the crosslinking degree and thereby the carbon yield of P4VP, which can be tuned by varying the feeding ratio (molar ratio of K_2_PtCl_4_ with respect to the pyridine ring). When the feeding ratio was 20%, Pt NP‐loaded MCMPs with oblate shapes and highly ordered hexangular structures consistent with the template BCP microparticles were successfully obtained (Figure 2b). Pores on the surface of MCMPs can be clearly observed from SEM images (Figure S7a, Supporting Information), indicating that they are open pores. A broken MCMP shows that the inner pore structure is the same as the surficial pore structure (Figure S7b, Supporting Information), which implies that the pores are vertically oriented cylindrical pores. EDX results (Table S1, Supporting Information) indicate that the relative atomic fraction of Pt/N is 75/100. The relative atomic fraction of Pt/N decreased to 30/100 with a decrease in the feeding ratio to 10%. There was no obvious difference in the morphology of the MCMPs when the feeding ratio decreased to 10% (Figure S7c, Supporting Information), and the oblate shape is clearly shown in the SEM image (Figure S7d, Supporting Information). When the feeding ratio was further decreased to 5% or lower, only fragments of mesoporous carbon were obtained, and no MCMPs formed when the feeding ratio was 1% or lower (Figure S8, Supporting Information). This result suggested that the introduction of Pt NPs plays a critical role in ensuring that the pre‐engineered shape and mesostructuring of BCP microparticles can be exactly inherited by MCMPs after carbonization.

### 2.3 Morphology and Structure of Pt NP‐Loaded MCMPs

**Figure 3:**
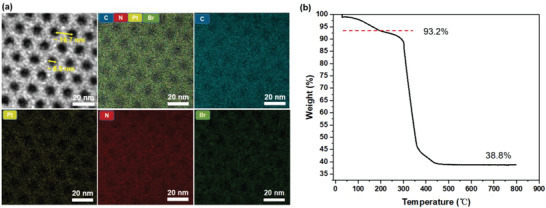
a) A dark field HRTEM image of a Pt NP‐loaded MCMP and corresponding element mapping images; b) The TGA curve of Pt NP‐loaded MCMPs in air with a temperature range of 30–800 °C.

The in situ growth process ahead of carbonization allows the generation of relatively uniformly sized Pt NPs, which can be clearly observed to be uniformly distributed in the carbon frame. High‐resolution TEM (HRTEM) images show that the diameters of Pt NPs are smaller than 5 nm (Figure 2c), with a crystal lattice of ≈2 Å (the inset of Figure 2c). The single‐crystal feature of Pt NPs was further confirmed by XRD with a main peak of the (111) crystal plane (Figure 2d). In addition, element mapping images show the uniform distribution of Pt and N atoms in the carbon matrix (**Figure** **3**a). The method also enables high density and high loads of Pt NPs in mesoporous carbon carriers. The TGA data indicate that the mass fraction of Pt is 41.6% when the feeding ratio of the precursor is 20% (Figure 3b).

**Figure 4:**
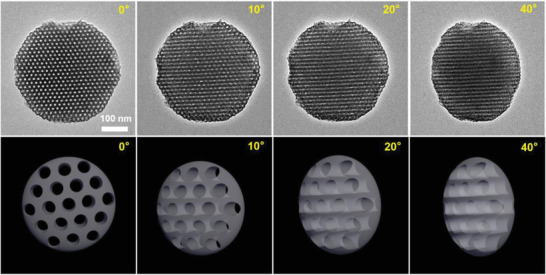
Tilt angle TEM images of a Pt NP‐loaded MCMP and corresponding 3D cartoons showing the orientation of pore pathways.

**Figure 5:**
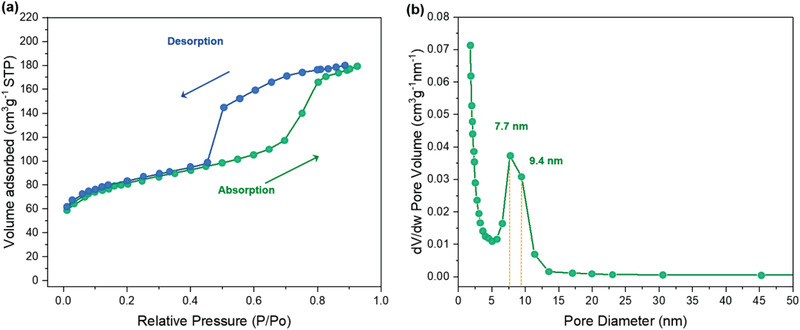
a) Nitrogen adsorption–desorption isotherms and b) pore size distribution of Pt NP‐loaded MCMPs.

This approach allows the preparation of MCMPs with ordered and uniform cylindrical mesopores. Their hexangular‐packed cylinder mesopores were confirmed by tilt angle TEM images (**Figure** **4**), and cylinder mesopores could be clearly observed from the longitudinal sections of cracked MCMPs (Figure S9, Supporting Information). The small‐angle XRD spectrum confirms the presence of an ordered mesostructure (Figure S10, Supporting Information), and the estimated periodic distance (*d*) determined from the equation 2*d*sin*θ* = *λ* is 15.5 nm, where *θ* belongs to the position of the strong peak at 2*θ* = 0.57°. In addition to anisotropic shapes and highly ordered and oriented pore paths, the MCMPs possess uniformly sized mesopores larger than 5 nm. From the HRTEM images, we can clearly see uniformly sized large mesopores with an average size of ≈8.5 nm, and the period distance is ≈16.7 nm (Figure 3a). In comparison with the template particles, slight decreases in the size of the mesostructures can be attributed to shrinkage during calcination. The pore diameter matched well with that of PS cylinders in template microparticles, and the wall thickness matched well with the distance between PS cylinders. Nitrogen adsorption–desorption isotherms were measured to investigate the porosities of the Brunauer–Emmett–Teller (BET) surface area (**Figure** **5**). The MCMPs have a type‐IV curve, confirming the mesoporous structure, and the BET surface area is calculated to be 292.2 m^2^ g^–1^. The size of mesopores measured by BET is consistent with that presented in HRTEM, and a narrow distribution of pore size is observed (Figure 5b). In addition, micropores smaller than 2 nm also exist, which can be mainly attributed to the removal of PDP.

In addition, the method enables fine tuning of the pore size by varying the molecular weight of PS‐*b*‐P4VP (**Table** **1**). As shown in Figure S11a,b (Supporting Information), microparticles with enlarged PS cylinders and increased distance of neighboring PS cylinders are obtained from PS_212_‐*b*‐P4VP_206_. As a result, Pt NP‐loaded MCMPs (Figure S11c,d, Supporting Information) with larger pores of ≈16 nm and thicker walls of ≈17 nm can be obtained.

**Table 1:** Size statistics of mesostructures of BCP microparticles and pore size and wall thickness of Pt NP‐loaded MCMSs

BCPs	d_PS_ ^a)^	l_P4VP_ ^b)^	d_pore_ ^c)^	t_wall_ ^d)^
PS_94_‐*b*‐P4VP_95_	10 nm	9 nm	8.5 nm	8.2 nm
PS_212_‐*b*‐P4VP_206_	20 nm	15 nm	16 nm	17 nm

^a)^ Diameter of the PS cylinder

^b)^ Distance between adjacent PS cylinders of BCP microparticles

^c)^ Pore size

^d)^ Wall thickness of Pt NP‐loaded MCMPs.

### 2.4 Universality of This Concept

**Figure 6:**
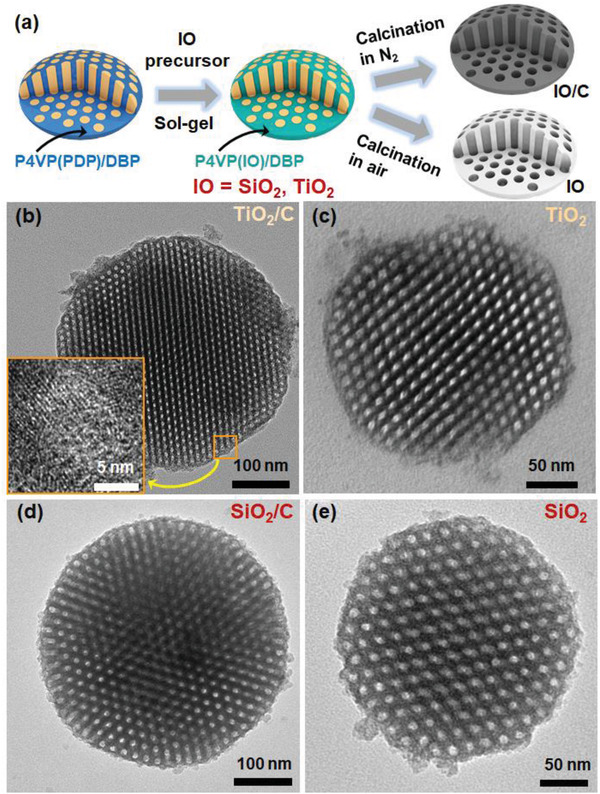
a) Schematic illustration of the synthesis route of IO or IO/C mesoporous microparticles; b,d) TEM images of TiO_2_‐loaded and SiO_2_‐loaded MCMPs, respectively; c,e) TEM images of TiO_2_ and SiO_2_ mesoporous microparticles, respectively.

This strategy is general and can be extended for the fabrication of a wide range of mesoporous microparticles with various compositions, such as metal and inorganic oxides (IOs) and their composites with carbon (**Figure** **6**a), because the reactive pyridyl moieties can complex not only with metals (e.g., Pt, Au, and Pd)^[^ ^19^ ^]^ but also with precursors of inorganic oxides.^[^ ^20^ ^]^ TiO_2_ is well known for its photocatalytic performance, and combining features of TiO_2_ with MCMPs should be of great interest. In this study, we show that our strategy enables the preparation of TiO_2_‐composited MCMPs and pure TiO_2_ mesoporous microparticles. Ethanol was used as the solvent for the absorption of citric acid‐titanium chelate. Interestingly, TiO_2_‐composited MCMPs with shape and pore structure similar to those of Pt NP‐loaded MCMPs were successfully obtained (Figure 6b). Their elemental composition was confirmed by EDX (Figure S12a, Supporting Information), and the crystal lattice of TiO_2_ was shown in the HRTEM image (inset of Figure 6b). To further investigate the morphology of TiO_2_, carbon was removed via secondary calcination at 700 ℃ in an air atmosphere for 2 h, and mesoporous microparticles of pure TiO_2_ were obtained (Figure 6c). In addition, TiO_2_ can be easily replaced by SiO_2_ (Figure 6d,e and Figure S12b, Supporting Information). Obviously, the color of SiO_2_‐composited MCMPs is black, while that of pure SiO_2_ mesoporous microparticles is white (Figure S13, Supporting Information). These results confirm the versality of this method.

## 3 Conclusion

In summary, we have developed a facile yet robust route that enables the synthesis of oblate mesoporous microparticles with almost *p6mm* structure, especially composite MCMPs. This route of decoupling neutral interface‐guided 3D‐CSA from self‐templated direct carbonization provides the possibility of first engineering BCP microparticles to obtain the unique morphology and mesostructures and subsequently selective crosslinking (and decoration) followed by carbonization of their continuous phase to form composite MCMPs without using any external carbon source. Many functional species, such as metal NPs and transition metal oxides, can be used as crosslinkers, which makes this method versatile. This approach, when coupled with Shirasu Porous Glass membrane emulsifying technology or droplet microfluidics, may enable superior control of the overall size uniformity of mesoporous microparticles over a wide range of 100 nm–10 µm. These composite MCMPs integrate features of anisotropic shape, highly ordered and oriented pore pathways, and uniform and tunable pore size, as well as functional inorganic decoration and *N*‐doping, which would find important applications in fuel cells, separation and heterogeneous catalysis.

## 4 Experimental Section

### Materials

PS_94_‐*b*‐P4VP_95_ (*M_w_*/*M_n_* = 1.08) and PS_212_‐*b*‐P4VP_206_ (*M_w_*/*M_n_* = 1.15) were purchased from Polymer Source, Inc. Poly(vinyl alcohol) (PVA, average *M_w_* = 13 000–23 000 g mol^–1^, 87–89% hydrolyzed) and K_2_PtCl_4_ (≥99.9%) were purchased from Aldrich. 3‐Pentadecylphenol (PDP, >90%), 1,5‐dibromopentane (DBP, 98%), citric acid (99%), ascorbic acid (AA, 99.995%), ammonia water (25–28 vol%), ethyl orthosilicate (TEOS, AR), and tetraisobutyl orthotitanate (98%) were purchased from Aladdin Biochemical Technology Co., Ltd. Dichloromethane (DCM) and EtOH were purchased from Shanghai Titan Scientific Co., Ltd. Materials were used without further purification except PDP. PDP was purified by recrystallization in *n*‐hexane.

### Preparation of BCP Microparticles by 3D‐CSA

PS‐*b*‐P4VP and PDP were dissolved in DCM to obtain 10.0 mg mL^–1^ solutions. Subsequently, these two solutions were mixed at a certain ratio and then stirred at room temperature for 12 h to form PS‐*b*‐P4VP (PDP)*_x_* supramolecules (the subscript *x* represents the stoichiometric ratio of PDP with respect to pyridine rings of P4VP). DBP was prediluted by DCM to 10 vol% before mixing with the supramolecule solution stirrer at room temperature for 4 h. Then, 0.1 mL of the mixed solution was emulsified with 1.0 mL PVA aqueous solution (5.0 mg mL^–1^) by using a membrane‐extrusion emulsification technique.^[^ ^13i^ ^]^ The emulsion was collected in an open vial (10 mL), and microparticles were generated after natural evaporation of DCM for 48 h at 30 °C. Finally, PVA was removed by centrifugal separation (14 000 rpm for 10 min).

### Decoration of Microparticles

1) Pt NP‐loaded composite microparticles. ≈1.5 mg of preformed microparticles was dispersed in 2 mL of a mixture of deionized water and ethanol (vol, 1:1). K_2_PtCl_4_ was dissolved in water at a concentration of 50 mg mL^–1^. A certain K_2_PtCl_4_ solution was added to the colloidal dispersion and stirred at room temperature for 12 h, followed by the addition of 3 times AA (with respect to the mole ratio of K_2_PtCl_4_) and stirring for 12 h again. Finally, the composite microparticles were purified by centrifugation at 1400 rpm for 10 min.
2) TiO_2_‐loaded composite microparticles. Tetraisobutyl orthotitanate (1.42 g, 5 mmol) was dissolved in 2.5 mL ethanol, and citric acid (1.05 g, 5 mmol) was dissolved in 5 mL ethanol. The citric acid solution was slowly dropped into the tetraisobutyl orthotitanate solution and stirred at 40 ℃ for 2 h to obtain a titanium citrate solution. Twenty microliters of titanium citrate solution were added to 2 mL ethanol solution containing ≈1.5 mg of preformed microparticles and then stirred for 12 h again. Finally, the composite microparticles were purified by centrifugation at 1400 rpm for 10 min.
3) SiO_2_‐loaded composite microparticles. ≈1.5 mg of the preformed microparticles was dispersed in 2 mL ethanol, followed by the addition of 3 µL TEOS and stirring for 12 h. Then, 40 µL ammonia water was diluted with 1 mL ethanol. Then, the two solutions were mixed together and stirred for 12 h. Finally, the composite microparticles were purified by centrifugation at 1400 rpm for 10 min.

### Calcination

For preparation of composite MCMPs, the composite microparticles were calcinated at 500 ℃ in a tube furnace with a nitrogen atmosphere for 4 h. For preparation of SiO_2_ mesoporous particles, the SiO_2_‐loaded composite microparticles were calcinated at 500 ℃ in an air atmosphere for 2 h. The heating‐up rate was 10 ℃ min^–1^.

### Characterization

The internal structure of the particles was observed by using a Hitachi HT7700 transmission electron microscope (TEM) operated at an accelerated voltage of 100 kV. Element mapping and Pt loading were measured by an energy dispersive X‐ray spectroscopy (EDX) accessory of the HRTEM. Elemental analysis of TiO_2_ and SiO_2_ was performed using an EDX accessory for TEM. High‐resolution TEM images and lattice and tilt angle images were obtained by using a Talos F200X high‐resolution TEM (HRTEM) operated at an accelerated voltage of 200 kV. The surface morphology of the particles was observed by using a Hitachi SU8010 scanning electron microscope (SEM) operated at an acceleration voltage of 1.0 kV (BCP microparticles) or 10.0 kV (Pt NP‐loaded MCMPs). Before TEM and SEM characterization, the samples were stained with iodine vapor for 1 h. The height profile of particles was measured by an SPM‐9700 scanning probe atomic force microscope (AFM). UV–vis spectra of PDP were detected by a TU‐1810 spectrophotometer. X‐ray diffraction (XRD) data were collected by a SmartLab‐SE X‐ray polycrystalline powder diffractometer with Cu K*α* radiation (40 kV, 45 mA, wavelength is 0.154 nm). The specific surface area and pore size were analyzed by using an ASAP 2420‐4 MP automatic specific surface area and porosity analyzer. The size distribution of the particles was characterized by dynamic light scattering (DLS, Malvern Zetasizer Nano ZS90). The hydrogen‐bonding, quaternization and coordination interactions were analyzed by Fourier transform infrared spectroscopy (FT‐IR, Bruker VERTEX 70).

## Conflict of Interest

The authors declare no conflict of interest.

## Supporting information

Supporting Information

## Data Availability

The data that support the findings of this study are available from the corresponding author upon reasonable request.
