# Supplementary material for: A Versatile 3D‐Confined Self‐Assembly Strategy for Anisotropic and Ordered Mesoporous Carbon Microparticles
Source: Adv Sci (Weinh). 2022 Jul 3;9(25):2202394. doi: 10.1002/advs.202202394 (PMC9443438; doi:10.1002/advs.202202394)
Supplement: Supplementary file 1 — Supporting Information [file ADVS-9-2202394-s001.pdf]

ADVANCED SCIENCE
Open Access

Supporting Information

for *Adv. Sci.*, DOI 10.1002/advs.202202394

A Versatile 3D-Confined Self-Assembly Strategy for Anisotropic and Ordered Mesoporous Carbon Microparticles

Mian Wang, Xi Mao, Jingye Liu, Bite Deng, Shuai Deng, Shaohong Jin, Wang Li, Jiang Gong, Renhua Deng and Jintao Zhu**

Supporting Information

A Versatile 3D-Confined Self-Assembly Strategy for Anisotropic and Ordered Mesoporous Carbon Microparticles

Mian Wang, Xi Mao, Jingye Liu, Bite Deng, Shuai Deng, Shaohong Jin, Wang Li, Jiang Gong, Renhua Deng, and Jintao Zhu**

Supporting Figures/Schemes:

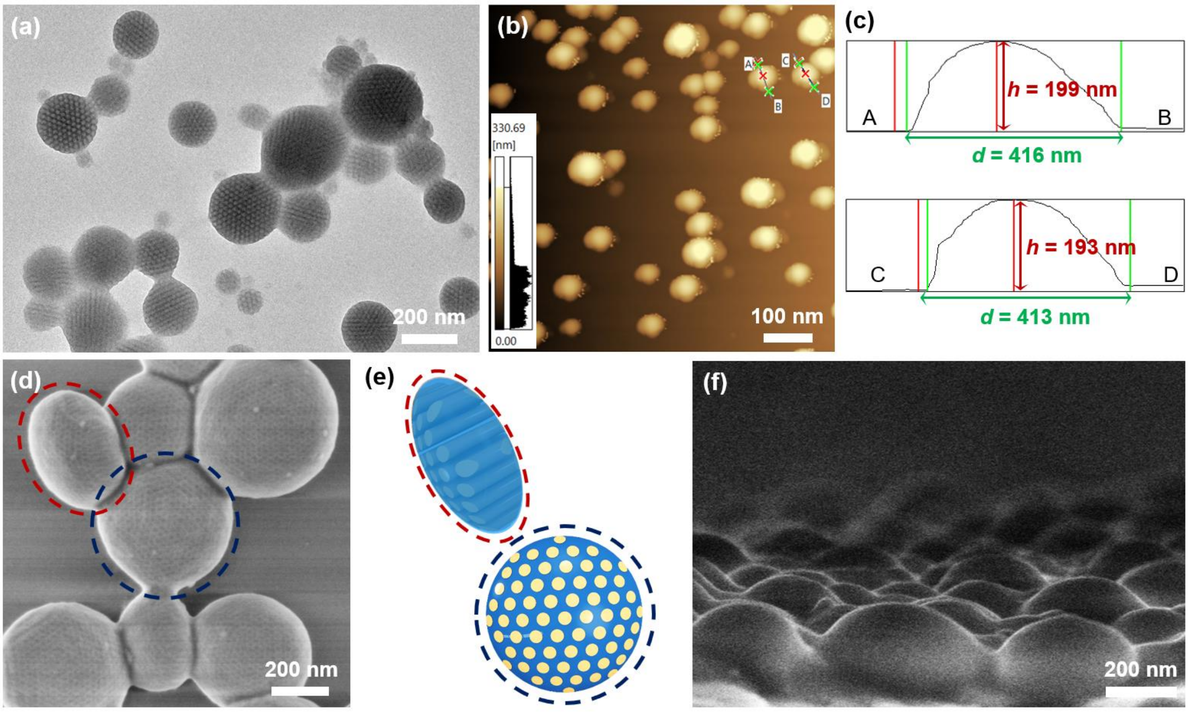

Figure S1. (a) TEM, (b) AFM, (d and f) top-view and side-view SEM-images of PS_{94}-*b*-$P4VP_{95}(PDP)_{0.6}/(DBP)_{0.4}$ microparticles; (c) height profile according to the AFM image; (e) 3D cartoons of particles from different angles.

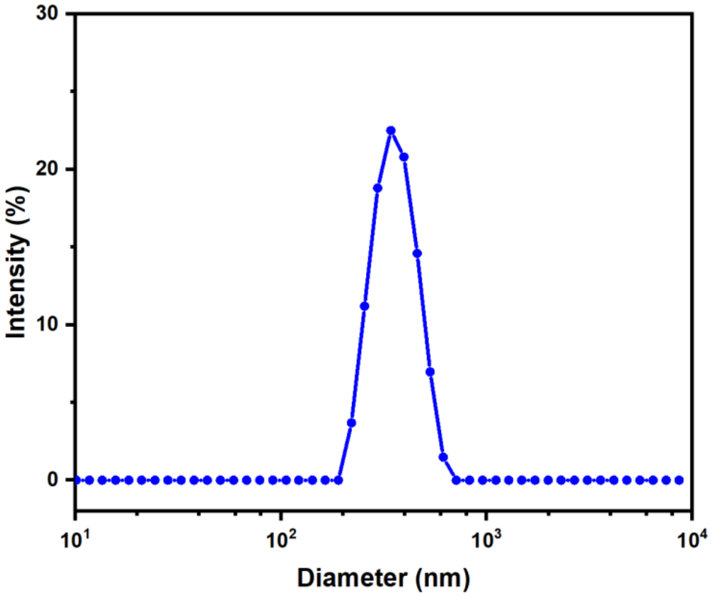

Figure S2. DLS data of PS_{94}-*b*-$P4VP_{95}(PDP)_{0.6}/(DBP)_{0.4}$ microparticles. The average equivalent diameter is 343.5 nm, and PDI = 0.078.

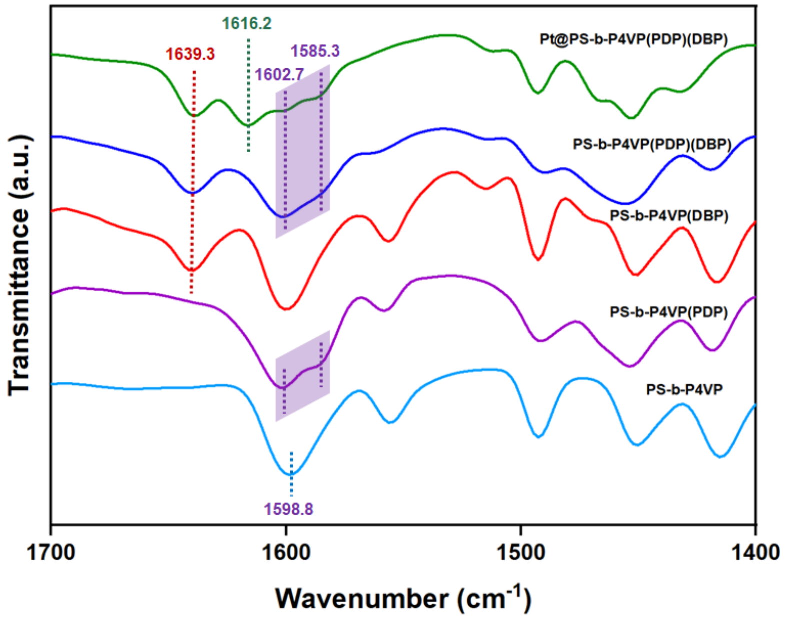

Figure S3. FT-IR spectra of microparticles with different compositions.

The peak at 1598.8 cm^{-1} belongs to the characteristic peaks of the pyridine ring in the nonbonded state. In the presence of PDP, a double peak (1602.7 cm^{-1} and 1585.3 cm^{-1}) instead of the single peak indicated the interaction of P4VP with PDP. On the other hand, the presence of DBP drives the shift of the peak to the higher frequency region: A new peak appears at 1639.3 cm^{-1}, which reveals particial pyridine groups interact with DBP. All the above peak shifts can be observed in the presence of both PDP and DBP. After "guest" exchange, we can see that the peak shifts induced by PDP become weak, suggesting that most PDP molecules are removed, while another new peak appears at 1616.2 $cm^{-1,}$ which can be ascribed to the coordination interactions between Pt and N atoms. [S1]

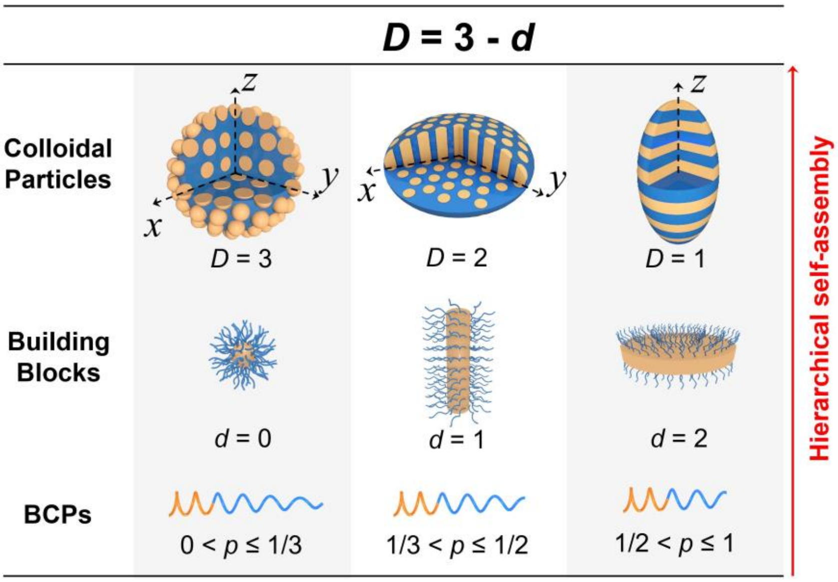

Scheme S1. Schematic illustration of neutral interface guided hierarchical self-assembly of BCPs under 3D soft confinement following the $D = 3 - d$ law, where d is predictable according to the packing parameter (p).

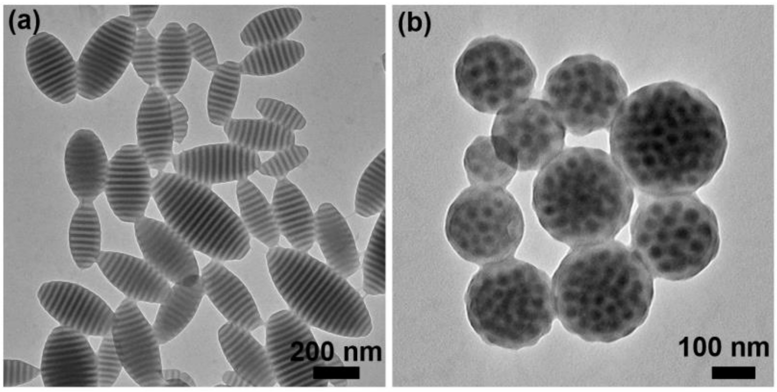

Figure S4. TEM images of PS_{94}-*b*-$P4VP_{95}$ microparticles ($D = 1$, $d = 2$) and PS_{490}-*b*-$P4VP_{171}$ microparticles ($D = 3$, $d = 0$).

BCPs	PS_m-*b*-$P4VP_n(PDP)_{x/n}$		
	ref. 13a, 13m	This work	ref. 13n
M_{PS}	9.8k ~ 10k	9.8k	50k
M_{P4VP}	10k	10k	13k
x/n	0	0.6	0.5
f_{PS}	0.5	0.26	0.61
**Morphology**	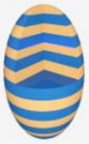	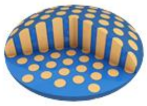	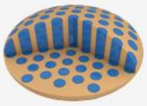
**Continuous Phase**	/	P4VP(PDP)	PS
**MCP Templates**	/	**Yes**	/

Scheme S2. Summary of anisotropic microparticles of PS-*b*-P4VP(PDP) compared with previous reports (f_{PS}: the volume fraction of the PS block).

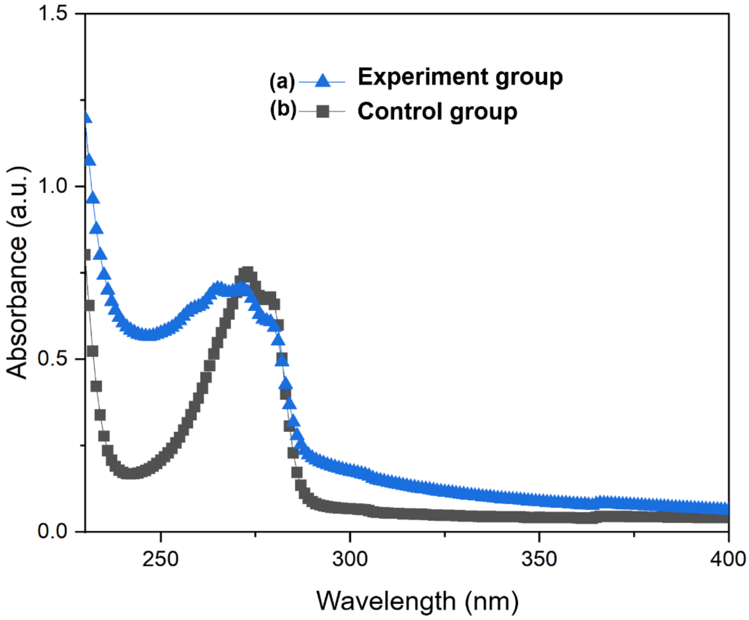

Figure S5. UV–vis spectra of (a) the supernatant of colloidial particle solution after compositing with Pt in water/ethanol (vol. 1:1) and (b) a neat PDP solution with the water/ethanol (vol. 1:1) mixture as the solvent.

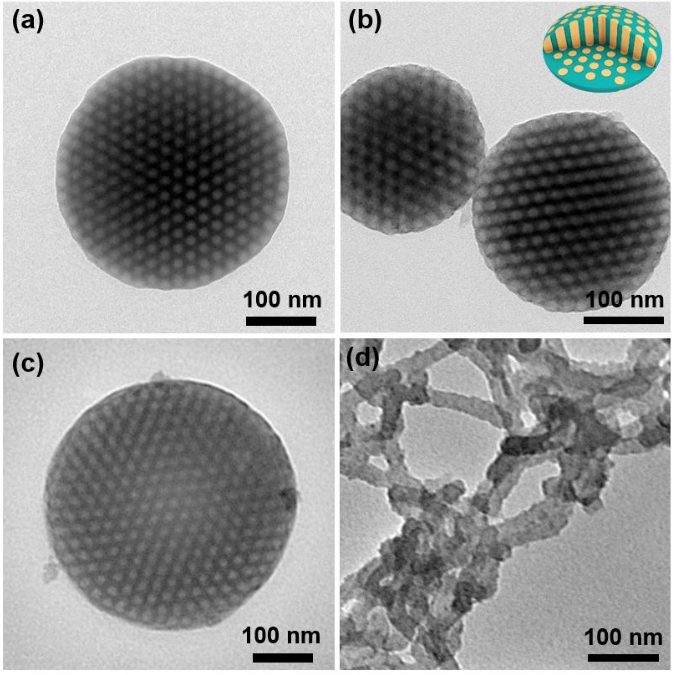

Figure S6. (a, b) TEM images of PS_{94}-*b*-$P4VP_{95}(PDP)_{0.6}/(DBP)_{0.4}$ microparticles before and after decoration with Pt NPs; (c, d) TEM images of PS_{94}-*b*-$P4VP_{95}(PDP)_{0.6}$ microparticles before and after decoration with Pt NPs.

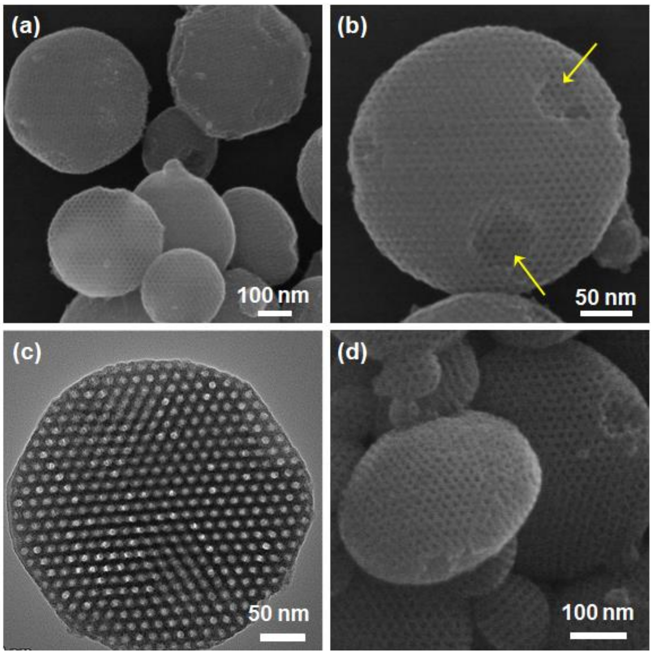

Figure S7. (a and b) SEM images of Pt NP-loaded MCMPs prepared with a feeding ratio of 20%; (c) TEM image and (d) SEM image of Pt NP-loaded MCMPs prepared with a feeding ratio of 10%.

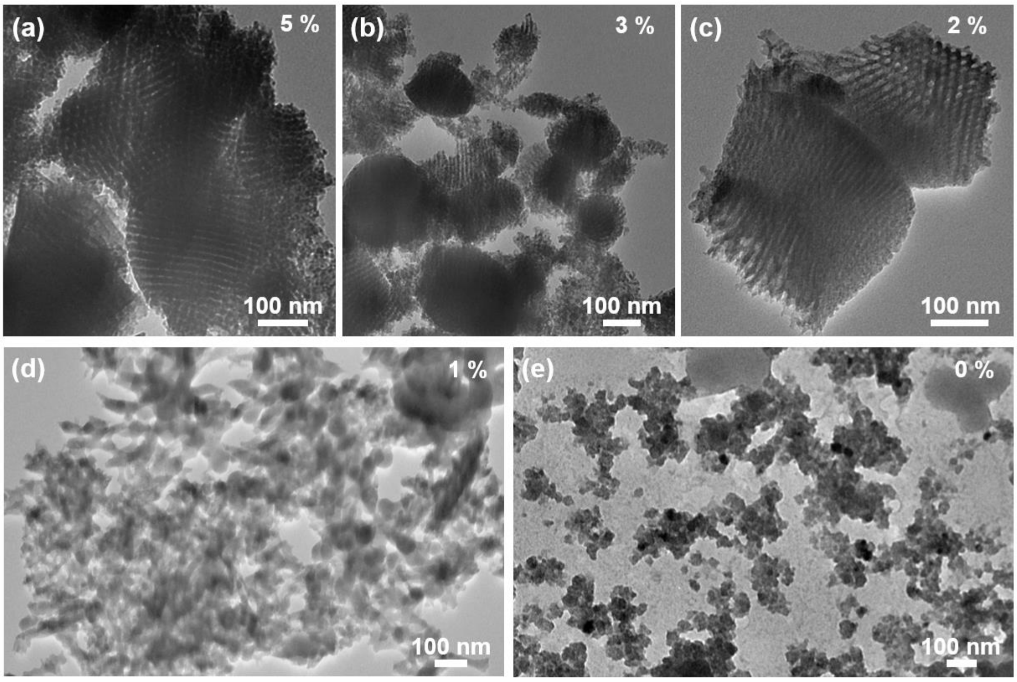

Figure S8. TEM images of Pt/C products derived from carbonized microparticles composting different Pt loading preads by varying feeding ratios (lablled upright).

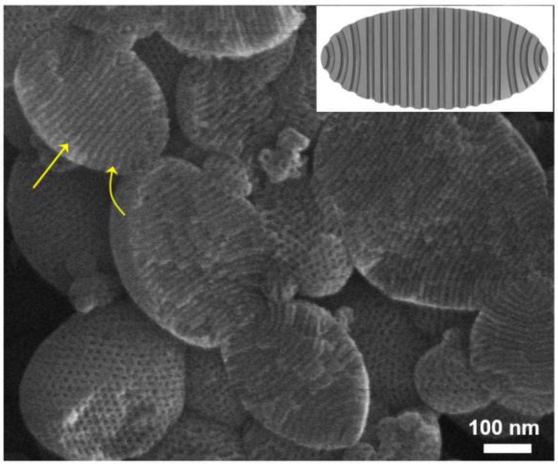

Figure S9. SEM image of longitudinal sections of fracture MCMPs (feeding ratio of 10%). The inset is a cartoon showing the cross-section of the microparticles. Not all the mesopores are straight, and their curvature increases from the centre to the edge, which matches the surface curvature of the particle well.

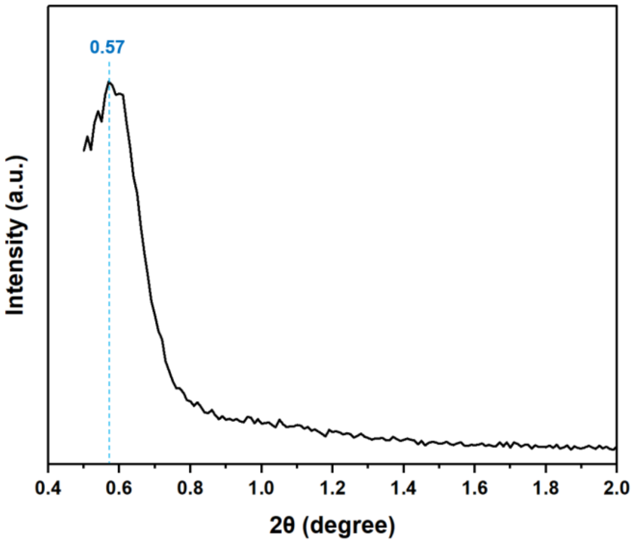

Figure S10. Small-angle XRD spectrum of Pt NP-loaded MCMPs.

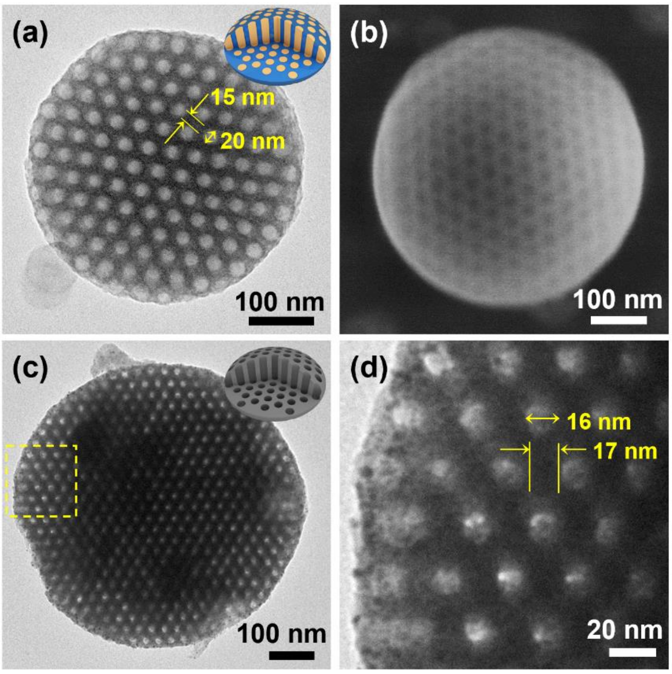

Figure S11. (a) TEM and (b) SEM images of a colloidal particle of PS_{212}-*b*-$P4VP_{206}(PDP)_{0.5}/(DBP)_{0.4}$, and (c, b) TEM images of a corresponding Pt NP-loaded MCMP.

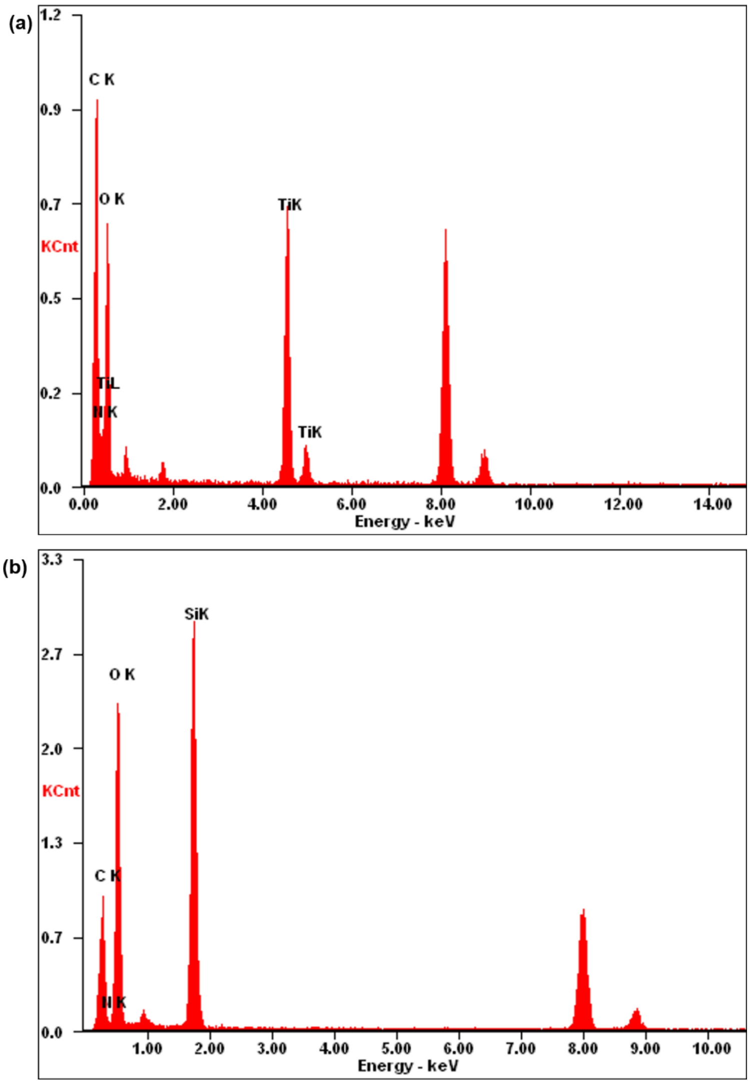

Figure S12. EDX elements of (a) TiO_2-loaded MCMPs and (b) mesoporous particles of SiO_2 on a carbon film.

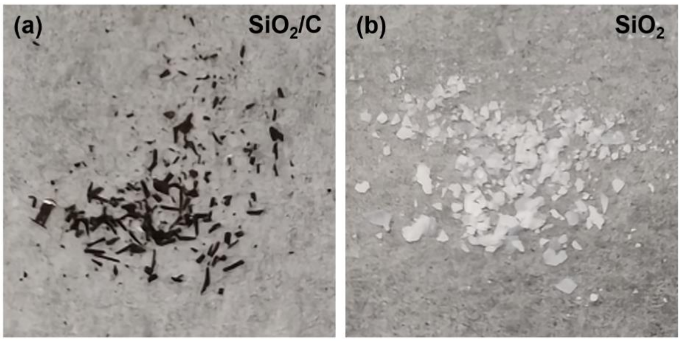

Figure S13. Photographs of powders of (a) SiO_2/C and (b) SiO_2 mesoporous microparticles.

Supporting Table:

Table S1. EDX element fraction of Pt NP-loaded MCMSs.

Feeding ratio	Element	C	N	Pt	Br
10%	Atomic Fraction (%)	95.40	**3.42**	**1.04**	0.14
	Mass Fraction (%)	81.35	3.40	14.47	0.79
20%	Atomic Fraction (%)	93.63	**3.55**	**2.68**	0.15
	Mass Fraction (%)	65.83	2.91	30.57	0.69

Reference:

[S1] A. Drelinkiewicz, J.W. Sobczak, E. Sobczak, M. Krawczyk, A. Zieba, A. Waksmundzka-Góra, *Mater. Chem. Phys.* **2009**, *114*, 763–773.
